# Functional shape of the proximal radioulnar joint: anatomical characterization through Alpha and Beta Angles

**DOI:** 10.1016/j.xrrt.2026.100695

**Published:** 2026-02-06

**Authors:** Aurelien Traverso, Valeria Vismara, Simone Cassin, Elias Kaleb Rojero-Gil, Erica Lante, Marius Andrei Sainiuc, Andrea Zagarella, Pietro Simone Randelli, Paolo Arrigoni

**Affiliations:** a Department of Orthopaedics and Traumatology, Centre Hopitalier Universitaire Vaudois (CHUV), Lausanne, Switzerland; b Scuola Di Specializzazione in Ortopedia e Traumatologia Università Degli Studi Di Milano, Milan, Italy; c Hospital Ángeles Pedregal, Ciudad de México, Mexico; d Noble's Hospital, Isle of Man, UK; e U.O.C. Radiodiagnostica, Azienda Socio Sanitaria Territoriale Centro Specialistico Ortopedico Traumatologico Gaetano Pini-CTO, Milan, Italy; f Laboratory of Applied Biomechanics, Department of Biomedical Sciences for Health, Università Degli Studi Di Milano, Milan, Italy; g U.O.C. 1° Clinica Ortopedica, ASST Centro Specialistico Ortopedico Traumatologico Gaetano Pini-CTO, Milan, Italy; h Research Center for Adult and Pediatric Rheumatic Diseases (RECAP-RD), Department of Biomedical Sciences for Health, Università Degli Studi Di Milano, Milan, Italy; i Clinica Ortopedica, Azienda Socio Sanitaria Territoriale Centro Specialistico Ortopedico Traumatologico Gaetano Pini-CTO, Milan, Italy

**Keywords:** Proximal radioulnar joint, Forearm, Radial head, Ulnar notch, Joint stability, Anatomical variation, Computed tomography (CT), Angular measurement

## Abstract

**Background:**

The proximal radioulnar joint (PRUJ) plays a critical role in forearm rotation through the articulation between the radial head (RH) and the ulnar notch. While previous studies have emphasized the importance of congruency for joint stability, limited data exist on anatomical variations in healthy individuals and their potential biomechanical implications. This study aimed to describe novel anatomical parameters of the PRUJ.

**Methods:**

A retrospective analysis of 104 elbow computed tomography scans from patients without elbow instability or deformities was performed. Measurements were taken on standardized axial computed tomography slices using two novel angular metrics: the Alpha and Beta Angle. Linear regression was used to assess the association between these angles.

**Results:**

The mean Alpha Angle was 139.82° (standard deviation 10.08°), while the mean Beta Angle was 81.25° (standard deviation 11.04°), both showing high interindividual variability. A statistically significant negative correlation was found between Alpha and Beta Angles (β = −0.53, 95% confidence interval: −0.72 to −0.34, *P* < .0001).

**Discussion:**

A larger Alpha Angle implies a less concave notch and reduced bony constraint of the RH, increasing the reliance on ligamentous structures, particularly the annular ligament, for joint stability. Conversely, a higher Beta Angle, indicating increased RH coverage, may confer greater intrinsic stability.

**Conclusion:**

The study identifies a significant inverse relationship between the Alpha and Beta Angles of the PRUJ, supporting the concept that ulnar notch morphology affects RH coverage and forearm stability. These reference values in healthy elbows may serve as a baseline for future comparisons in pathological conditions and inform the diagnosis and management of joint instability and degeneration.

Radius and Ulna function together with the proximal radioulnar joint (PRUJ), the distal radioulnar joint, and the interosseous membrane that runs between them.^16^^,^^18^

RUJ plays a crucial role in forearm mechanics, enabling pronation and supination through the interaction of the radial head (RH) with the ulnar notch.^10^^,^^24^ The shape of the RH varies from elliptical to circular.^1^ Proper alignment and stability of the RH within the radial notch are essential for smooth joint function.^11^ The annular ligament provides stability during rotational movements.^11^^,^^14^^,^^21^

Early anatomical studies have described the radial height and radial width of the lesser sigmoid notch, but not in relationship to the RH. More recent literature based on three-dimensional modeling of the lesser sigmoid notch has highlighted how the lesser sigmoid notch is neither straight nor uniform, but angulated in the transverse plane in an antero-radial direction and in the sagittal plane, being most shallow distally. Furthermore, a high variability, up to 39%, was found in the angle comprised by the line intersecting the anterior and posterior edge of the sigmoid notch and the transverse plane of the ulna, passing through the coronoid tip.^23^ Even though the relationship between the coronoid and the RH has been recently described,^22^ The anatomic variability of the PRUJ has not been thoroughly assessed. While prior studies have highlighted the importance of anatomic congruity to maintain joint stability^6^^,^^13^^,^^14^ and preventing degenerative changes, and the influence of pronation and supination on the PRUJ,^12^^,^^19^ a detailed analysis of anatomical parameters remains limited.

The aim of the study is to describe the shape and congruency of the radial facet of the PRUJ.

## Materials and Methods

A retrospective analysis was conducted using Computed Tomography (CT) scans from patients with intact PRUJ anatomy, selected from hospital imaging databases. The study included individuals with mature skeletons and stable elbows, as determined by the absence of significant instability, posterolateral rotatory instability (PLRI),^13^ or abnormal clinical-radiographic signs such as Symptomatic Minor Instability of the Lateral Elbow.^2^^,^^3^ Exclusion criteria involved articular fractures of the elbow joint, significant deformities, rheumatoid arthritis, and all conditions compromising PRUJ anatomy.

Institutional approval of the study protocol was obtained before the study began (Resolution n° 138 dated 18 March 2021- 233_2021) and conducted according to the Declaration of Helsinki.

CT imaging was conducted using a standardized protocol, ensuring high-resolution axial images. RH and radial notch structures were isolated in cross-sectional views. All measurements were performed on the same axial CT cut that allowed the most proximal complete view of the RH. CT scans were acquired using a 64-slice CT scanner (Revolution EVO, GE Healthcare). The patient was positioned prone on the scanning table with the affected elbow overhead at a 45° flexion angle and a helical scan was obtained from the distal humerus, above the epicondyles, to the proximal radius and ulna, below the radial tuberosity (130 mm scan range, 100 mm field of view). Scans were acquired using the following technical parameters: 100 kVp, 80 mAs, 0.531 pitch, 0.625 mm slice thickness and interval, 512 × 512 pixels image matrix. Bone kernel reconstructions (1 mm slice thickness) on the axial, coronal, and sagittal planes were assessed.

To assess precise metrics for the evaluation of the biomechanical efficiency of the PRUJ, two anatomical parameters were measured and quantified.

**Figure 1:**
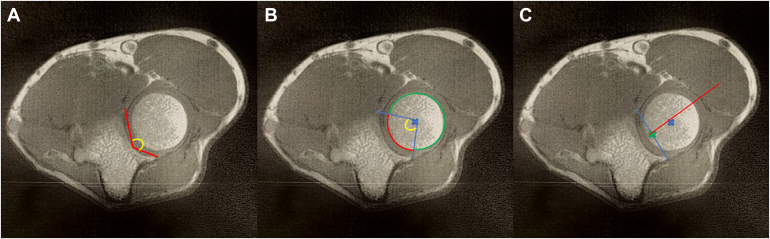
Key morphological parameters of the PRUJ. (1A) Alpha Angle, angle between lines from the anterior/posterior margins to the deepest point of the radial notch. (1B) Beta Angle, angle from the Center of the radial head to the anterior and posterior edges of the notch. *PRUJ*, proximal radioulnar joint.

The Alpha Angle, defined as the angle formed between the line from the most anterior profile of the radial notch and the deepest point of the radial notch, and the line from the most posterior profile of the radial notch and the deepest point of the radial notch^9^ (Fig. 1*A*).

The Beta Angle measures the angle formed by the two lines originating from the center of the RH and passing through the most anterior and posterior extremities of the radial notch^3^ (Fig. 1*B*).

All measurements were conducted by two orthopedic surgeons. A multivariate linear regression was performed to assess the relation between Alpha Angle (°) and Beta Angle (°). Data were checked for multicollinearity with the Belsley-Kuh-Welsch technique. Heteroskedasticity and normality of residuals were assessed, respectively by the Breusch-Pagan test and the Shapiro-Wilk test. A *P* value <.05 was considered statistically significant. The Newey-West correction for heteroskedasticity was applied. Statistical analysis was performed with EasyMedStat (version 3.38).

## Results

A total of 104 consecutive elbows were analyzed, with ages ranging from 16 to 93 years and an average age of 50 years. Among the samples, 34.6% were female, and the right limb was analyzed in 56.7% of the cases.

Each of the parameters was evaluated in all the 104 CT scans independently by the different operators at different time points with at least 15 days of interval in order to minimize the impact of both intra and interobserver biases without substantial differences between the assessments. All the parameters revealed significant variability across the patient population (Table I).

**Table I:** Measures of the CT PRUJ parameters.

Measurement	Mean	Median	Standard deviation	Range
Alpha angle	139.82°	140.83°	10.08	101.9°-163.11°
Beta angle	81.25°	81.65°	11.04	56.5°-122.95°

*CT*, computed tomography; *PRUJ*, proximal radioulnar joint.

Values are reported as mean, median, and range. The Alpha and Beta Angles are expressed in degrees (°).

No significant differences in sex and age distribution were observed. A linear regression analysis was conducted to assess the relationship between patient age and the Alpha Angle. The results showed a mild positive association, with Alpha Angle tending to increase slightly with advancing age. Despite this trend, the high variability among individual values suggests that age alone is not a strong predictor of Beta Angle in this population. As the Alpha Angle increases, the ulnar facet of the PRUJ tends to become more curved in the anteroposterior plane and congruent in morphology, which may augment its capacity to constrain the RH effectively.

**Figure 2:**
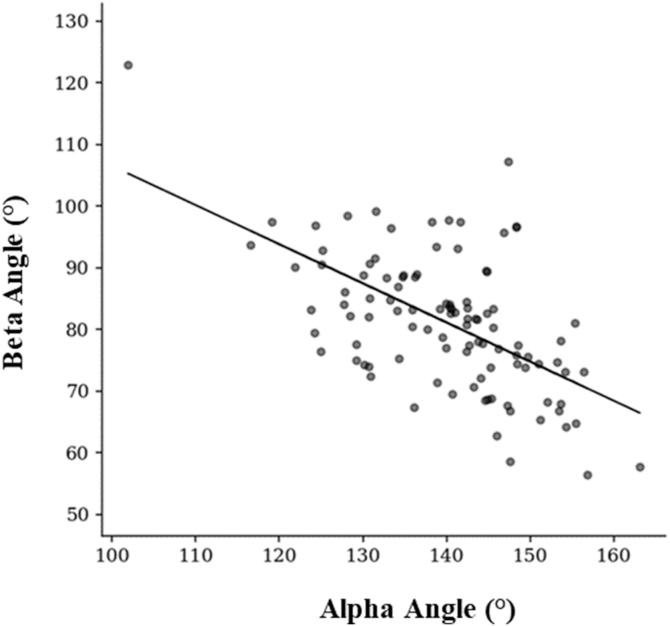
Negative correlation between the Beta Angle and the Alpha Angle. Each point represents an individual measurement, with the regression line illustrating the inverse relationship between these parameters. As the Alpha Angle increases, there is a corresponding decrease in the Beta Angle.

For group comparisons, multivariate linear regression revealed a significant negative relationship between the Alpha Angle and Beta Angle, with a regression coefficient of β = −0.53 [95% confidence interval: −0.72, −0.34], *P* < .0001 (Fig. 2). The multivariate linear regression analysis confirmed this relationship, indicating that the higher the Alpha Angle the lower the Beta Angle, meaning that the higher the ulnar opening angle, the lower the corresponding RH coverage; higher Alpha Angle leads to reduced bony coverage of the RH. This result is consistent with other research by Ha et al^9^ and Kim et al,^12^ who both highlighted similar relationships in elbow joint congruence.

The findings of this study provide support for the initial objective.

## Discussion

This study aimed to present brand new anatomical parameters of the PRUJ in a cohort of 104 healthy elbows. These parameters confirm the high variability of the position of the RH in respect to the lesser sigmoid notch.

The Alpha Angle assessments indicate a relatively consistent distribution even in the presence of occasional outliers. The Alpha Angle is an important indicator of joint alignment and can help to assess potential sources of instability in the PRUJ. This finding aligns with previous research by Kim et.^12^ Additionally, the Beta Angle measures suggest a substantial variability across cases, especially in the higher range of the angle. The Beta Angle is crucial for understanding the stability of the RH during forearm rotation, which is often compromised in pathological conditions. These findings are consistent with those of Bonnevialle, who noted variations in patients with lateral elbow pain, particularly in the context of joint degeneration or fracture sequelae.^7^

Our findings provide valuable insights into the interplay between these variables described, highlighting significant associations and the absence of certain expected correlations how the higher the ulnar opening angle the lower the corresponding is RH coverage. The high number of healthy subjects included in the study could allow to use these values as reference for comparison with pathological conditions. As the Beta Angle increases, the RH benefits from greater osseous coverage, resulting in enhanced containment and intrinsic stability. Conversely, a larger Alpha Angle is associated with reduced containment of the RH, thereby increasing reliance on soft tissue structures for joint stability. In particular, the stability of the RH within the PRUJ is primarily maintained by the annular ligament, which provides anterior closure of the articulation.

The inverse relationship between the Alpha Angle and the Beta Angle might reveal a potential impact on joint kinematics and pathology. The interaction between these two angles is crucial, as a reduced RH coverage can lead to greater movement and increased stress on the joint.^20^ Variations in elbow joint congruence may influence the extent of bony coverage, which is especially relevant in conditions such as elbow instability^11^^,^^17^ and other pathological conditions where joint congruence and coverage are essential to preserving joint function.^7^ When bony congruity is lacking, capsular and ligamentous structures need to provide higher stability, and lower stresses can lead to symptomatic instability. In shoulder, in instability settings, as the glenoid bone loss increases, the chances of recurrent dislocations following atraumatic events increase.^5^ In the elbow, the annular ligament keeps the RH centered into the lesser notch and stiffening either anteriorly or posteriorly according to the movement of the forearm.^8^ Elongation of this structure, following repetitive stresses in varus and pronation, in a patient with a less congruent joint could promote the development of symptomatic minor instability.^4^

On the other side of the spectrum, these angles could allow to better describe the progressive development of osteophytes in the stiff and arthritic elbow.^15^ An osteophyte on the posterior aspect of the lesser sigmoid notch, in fact, is often found in hypertrophic arthritic elbows, in up to 81% cases. This osteophyte would increase the Beta Angle and thereby increase the RH coverage, giving rise to a loss in prono-supination motion. The formation of this posterior osteophyte could be associated with a progressive relaxation of the capsuloligamentous structures, as it happens in the humeral head in the initial stages of arthritis.^5^ While the glenoid is much more flat then the lesser sigmoid notch, in the first case the progressive posterior displacement of the Humeral head leads to the development of a biconcave glenoid,^22^ it would seem that the lesser ulnar notch tries to compensate to this posterior displacement of the RH with excessive bone formation, at the expense of movement. A RH osteophyte was also found in 46% patients.^15^ The combination of these features could further decrease the patient range of motion. These findings are supported also by the fact that there was a mild positive association between the Beta Angle and advanced age, with Beta Angle tending to increase slightly with advancing age. Despite this trend, the high variability among individual values suggests that age alone is not a strong predictor of Beta Angle in this population.^3^^,^^12^

These findings add to the expanding body of knowledge on joint biomechanics, offering a foundation for better diagnostic and therapeutic approaches. They also carry significant implications for understanding forearm anatomy.

This work has some limitations. The position of the forearm was not consistent in all measurement: Kim et al show how the PRUJ is more congruent in supination rather than in pronation. Despite this being a very interesting finding, often, CT scan performed in the emergency room may not take into consideration the forearm position at the expense of patient comfort. Our cohort thereby displays a heterogeneous group of individuals in which both pronation, neutral, and supination positions are accounted for, allowing a broader use of the values described. Additionally, all CT scans were acquired with the elbow in extension, which may not fully represent joint relationships during functional positions such as flexion. The study population consisted solely of patients without structural elbow impairments, limiting the generalizability of these findings to pathological conditions; for this purpose, further studies on specific elbow pathologies will be necessary. Moreover, patients with overt intra-articular fractures were excluded from the analysis, minimizing the confounding effect of hemarthrosis, which could otherwise alter the relative positioning of the RH. Moreover, the retrospective nature of the analysis precludes causal inferences regarding the observed relationships, a limitation that is mitigated by the four different assessments.

Further investigations should explore these anatomical relationships in individuals with elbow pathologies, such as ligamentous injuries or degenerative conditions, to determine whether the observed associations hold in diseased states or if significant differences are found. Additionally, incorporating advanced imaging modalities, such as three-dimensional kinematic analyses, may enhance our understanding of how these parameters interact dynamically during motion.

## Conclusion

This study describes anatomical structures of the PRUJ and highlights a significant negative relationship between the Alpha Angle and the Beta Angle, emphasizing the biomechanical impact of joint congruence on bony coverage. The more the radial notch is extended anteroposteriorly, the more it encapsulates the RH.

## Disclaimers:

Funding: No funding was disclosed by the authors.

Conflicts of interest: The author, their immediate family, and any research foundation with which they are affiliated have not received any financial payments or other benefits from any commercial entity related to the subject of this article.
